# Short-Term Effects of Structured Physical Activity With or Without Dietary Counselling in Early-Stage Chronic Kidney Disease Managed in Primary Care: A Non-Randomised Controlled Study

**DOI:** 10.3390/jcm15083169

**Published:** 2026-04-21

**Authors:** Lorena Bosnar Zelenika, Dragana Tišma, Tamara Ciko, Pero Hrabač, Ivana Vuković Brinar, Valerija Bralić Lang

**Affiliations:** 1 Zagreb Health Centre—Centar, Runjaninova 4, 10000 Zagreb, Croatia; lorena.bosnar@gmail.com (L.B.Z.); tisma333@yahoo.com (D.T.); szp@dzz-centar.hr (T.C.); 2 Department of Kinesiology Recreation and Kinesiotherapy, Faculty of Kinesiology, University of Zagreb, 10000 Zagreb, Croatia; 3 Department of Medical Statistics, Epidemiology and Medical Informatics, “Andrija Stampar” School of Public Health, School of Medicine, University of Zagreb, 10000 Zagreb, Croatia; pero.hrabac@mef.hr; 4 Department of Nephrology, Art. Hypertension, Dialysis and Transplantation, University Hospital Centre Zagreb, School of Medicine, University of Zagreb, 10000 Zagreb, Croatia; ivbrinar@gmail.com; 5 Family Medicine Specialist Office, 10000 Zagreb, Croatia; 6 Department of Family Medicine, “Andrija Stampar” School of Public Health, School of Medicine, University of Zagreb, 10000 Zagreb, Croatia

**Keywords:** chronic kidney disease, Croatia, dietary counselling, non-randomised controlled study, physical activity, primary healthcare

## Abstract

**Background/Objectives:** To evaluate the short-term effects of structured physical activity (PA), alone or combined with dietary counselling, in early-stage chronic kidney disease (CKD) patients managed in primary healthcare (PHC). **Methods**: This non-randomised controlled study was conducted in Croatia from 1 September to 30 November 2025. Ninety adults aged 40–75 years with early-stage CKD were allocated to three groups: structured PA, combined PA and dietary counselling, or control. Interventions included kinesiologist-led PA and, in the combined group, dietitian-led Mediterranean/plant-based counselling. Outcomes included estimated glomerular filtration rate (eGFR), urinary albumin-to-creatinine ratio (ACR), cardiometabolic risk factors, behavioural measures, quality of life, and sleep quality. Statistical significance was set at *p* < 0.01. **Results:** Seventy-eight participants completed follow-up. Changes in eGFR did not differ between groups (*p* = 0.310). Mean ± standard deviation changes in ACR were −1.10 ± 6.37, −0.86 ± 2.88, and +1.18 ± 3.13 in the PA, combined, and control groups, respectively (*p* = 0.017, not meeting the prespecified significance threshold). Significant between-group differences were observed for selected patient-reported and PA outcomes, including emotional well-being, energy/fatigue, role limitations due to emotional problems, sedentary time, and total PA (all *p* ≤ 0.006). **Conclusions:** Structured PA, with or without dietary counselling, improved PA behaviour and selected patient-reported outcomes in early-stage CKD managed in PHC but did not demonstrate significant short-term effects on kidney-related outcomes. These findings support the feasibility of integrating lifestyle-oriented interventions into PHC as part of integrated CKD care, while larger, longer-term studies are needed.

## 1. Introduction

Chronic kidney disease (CKD) affects nearly 850 million people worldwide [1], including approximately 300,000 in Croatia [2]. It is a major under-recognised public health problem, with low awareness [3] and a projected rise to the fifth leading cause of death by 2040 [4].

Kidney function and damage are assessed using serum creatinine (sCr), estimated glomerular filtration rate (eGFR), and urinary albumin-to-creatinine ratio (ACR), which are central to CKD detection and risk stratification. Kidney Disease: Improving Global Outcomes (KDIGO) integrates these parameters in the G–A heat map to indicate the relative risk of renal or cardiovascular events [3].

Early CKD stages (G1–G3b) often remain undiagnosed, delaying timely intervention. The annual rate of eGFR decline varies [5] and can be accelerated by comorbidities such as diabetes mellitus and hypertension [6]. Patients with CKD, particularly those with stage G3 disease and more advanced stages, often have reduced physical function and lower levels of physical activity (PA) [7], which have been associated with higher all-cause mortality [8] and faster kidney function decline. This is unsurprising, as PA counselling and exercise prescription are not yet routinely integrated into CKD care [9].

CKD progression may be modified through lifestyle interventions alongside pharmacotherapy [10]. Given the high prevalence of early-stage CKD and limited access to specialist nephrology care, timely management in primary healthcare (PHC) is especially important. Family physicians (FPs) play a central role in early detection, risk stratification, and longitudinal management [11], while holistic CKD care emphasises non-pharmacological strategies such as a healthy diet, regular PA, smoking cessation, and weight control [3]. KDIGO recommends reassessing these modifiable risk factors every 3–6 months [3].

Contemporary CKD care increasingly emphasises integrated, patient-centred, multidisciplinary management. According to KDIGO, CKD care should include timely referral, regular follow-up, self-management support, and coordinated communication between primary and specialist care, although the optimal model varies with CKD severity and risk of progression. Various CKD care models have been described internationally [12,13,14,15,16], including programmes for patients approaching kidney failure and starting dialysis, highlighting the broader importance of coordinated care across the CKD continuum [17]. Multidisciplinary CKD care relies on complementary professional roles, with increasing recognition of the contribution of nephrology nurses to comprehensive CKD care [18,19]. Although team-based frameworks and lifestyle-oriented programmes are particularly relevant to PHC, both practical guidance and evidence for their implementation in early-stage CKD remain limited.

In CKD, regular PA has been associated with improvements in physical function, cardiovascular risk factors, and health-related quality of life (HRQoL). KDIGO therefore recommends PA compatible with cardiovascular health, individual tolerance, and level of frailty [3]. Even modest increases in PA may improve blood pressure (BP), physical and mental functioning, and overall HRQoL [7,20], while possible effects on eGFR remain uncertain and inconsistent [21,22], partly because of small and heterogeneous studies. CKD is also frequently accompanied by reduced functional reserve and frailty, particularly in older adults and those with multimorbidity, making frailty assessment increasingly relevant for risk stratification, shared decision-making, and care planning [3,23,24].

CKD progression also affects nutritional needs and increases the risk of metabolic and nutritional disorders. Current guidance increasingly emphasises overall dietary patterns rather than single nutrients, including a Mediterranean-style diet and referral to a dietitian or accredited nutrition provider when possible [3,25]. Combined PA and dietary interventions may also influence key risk factors for CKD progression, including BP, albuminuria, body mass index (BMI), and HRQoL [22].

Evidence on the effects of structured PA and dietary interventions in early-stage CKD, particularly on eGFR, remains limited. Interventions delivered in PHC may therefore offer an opportunity for earlier risk-factor modification and potential delay of disease progression [3,26].

The primary objective of this study was to evaluate the short-term effects of structured PA, delivered alone or with dietary counselling in PHC, on kidney-related outcomes, specifically eGFR and ACR, in adults with early-stage CKD. Secondary objectives were to assess cardiometabolic, behavioural, and patient-reported outcomes, and to explore whether the combined intervention was associated with greater changes than PA alone or usual care.

## 2. Materials and Methods

### 2.1. Study Design and Setting

This manuscript reports a prespecified analysis of three study groups from a larger prospective, nonrandomised, controlled interventional study conducted as part of a doctoral dissertation. The study was carried out in a PHC setting at Health Centre Zagreb—Centar (HCZC), Croatia, between 1 September and 30 November 2025.

The larger study comprised four parallel study groups. However, the present analysis includes only three prespecified groups: the PA intervention group, the combined PA and dietary counselling group, and the control group. Findings from the fourth study group, which received dietary counselling only, will be analysed separately and reported in the full doctoral dissertation.

Given the nonrandomised design, the study was reported in accordance with the Transparent Reporting of Evaluations with Nonrandomised Designs (TREND) statement, while intervention components were described in line with the Template for Intervention Description and Replication (TIDieR) checklist to improve transparency and reproducibility.

### 2.2. Participants and Eligibility Criteria

Participants were adults aged 40–75 years with early-stage CKD (stages G1–G3b) managed in PHC. Inclusion criteria were the ability to participate in the study procedures and complete the planned follow-up. Exclusion criteria were a life expectancy of less than 6 months, active malignancy, pregnancy, disability preventing participation in the intervention, or severe mental illness likely to affect adherence to the study protocol.

### 2.3. Recruitment, Sampling, and Group Allocation

Participants were recruited through 10 family medicine practices (FMPs) affiliated with HCZC. A systematic sampling approach was used, whereby every fifth eligible patient with early-stage CKD listed in alphabetical order within each FMP was approached for participation. If a patient declined participation, the next eligible patient on the list was invited.

The larger study was designed to include 120 participants, with 12 participants recruited from each FMP and allocated across four study groups. The present analysis includes 90 participants from three prespecified groups: the PA intervention group, the combined PA and dietary counselling group, and the control group. Group allocation followed a predefined random allocation procedure based on a list generated in SPSS (IBM SPSS Statistics for Windows, Version 25.0 (IBM Corp., Armonk, NY, USA)) [27].

### 2.4. Interventions

The intervention period lasted three months. Participants in the intervention groups received either a structured PA programme alone or a combination of structured PA and dietary counselling. The interventions were delivered at HCZC by trained healthcare professionals within the PHC setting.

#### 2.4.1. Physical Activity Intervention

The PA intervention consisted of a structured group exercise programme led by a kinesiologist. Groups included up to 15 participants. The programme combined aerobic, strength, balance, and flexibility exercises and was tailored to participants’ age and functional abilities. The intervention was designed to be consistent with World Health Organization recommendations for PA [28], aiming to support at least 150 min of moderate-intensity PA per week, or an equivalent volume of vigorous-intensity PA.

#### 2.4.2. Dietary Counselling Intervention

Participants in the combined intervention group also received dietitian-led dietary counselling. The counselling was individualised and focused on Mediterranean and plant-based dietary patterns. It comprised four sessions over 3 months: one initial 60 min session followed by three 30-minute follow-up sessions. Dietary intake in the combined intervention group was assessed at baseline and follow-up using 3-day food diaries [29], including one weekend day. These records were used to support individualised counselling, monitor adherence, and evaluate dietary quality.

#### 2.4.3. Control Group

Participants in the control group received usual care and did not participate in the structured exercise programme or receive dietary counselling during the 3-month study period.

No modifications to the intervention protocol were made during the study period.

### 2.5. Outcomes and Measurements

All participants were assessed at baseline and after the 3-month intervention period. The assessed outcomes included kidney-related parameters, cardiometabolic and clinical variables, behavioural risk factors, patient-reported outcomes, and dietary quality.

The primary outcomes for the present analysis were changes in kidney-related parameters, specifically eGFR and ACR. Secondary outcomes included sCr, cardiovascular risk factors (BP, lipid profile, and BMI), glycaemic parameters (fasting plasma glucose [FPG] and, if available, glycated haemoglobin [HbA1c]), KDIGO risk category, behavioural risk factors (smoking status, alcohol consumption, and level of PA), as well as HRQoL and sleep quality.

#### 2.5.1. Kidney-Related Outcomes

Kidney-related measurements included sCr, eGFR, ACR, and KDIGO risk category. eGFR was calculated using the 2021 Chronic Kidney Disease Epidemiology Collaboration (CKD-EPI) creatinine equation, as recommended by KDIGO [3]. ACR was measured in urine obtained from a first-morning midstream void sample, which is the preferred sampling method in adults [3]. KDIGO risk category was determined using the G–A classification based on eGFR and albuminuria categories.

#### 2.5.2. Cardiometabolic and Clinical Outcomes

Cardiometabolic and clinical outcomes included arterial BP, lipid profile, BMI, FPG, HbA1c, comorbidities, pharmacological treatment, and use of over-the-counter (OTC) products and supplements.

HbA1c was measured only when clinically indicated and was therefore analysed only in participants with diabetes mellitus. Information on comorbidities and ongoing pharmacological treatment at baseline and follow-up was extracted from medical records. Pharmacotherapy that could affect eGFR or ACR was not modified during the study period.

#### 2.5.3. Behavioural and Patient-Reported Outcomes

Smoking was categorised as 0 (non-smoker or quit >15 years ago) or 1 (current smoker or quit ≤15 years ago). Alcohol consumption was classified as 0 (non-drinker), 1 (low risk: <10 g/day for women and <20 g/day for men; 10 g corresponds approximately to 1 dL of wine, 0.5 L of beer, or one standard shot of spirits), and 2 (high risk: intake exceeding the recommended limits).

HRQoL, PA, and sleep quality were evaluated pre- and post-intervention using validated instruments: the Short Form (36) (SF-36) Health Survey [30], the International Physical Activity Questionnaire (IPAQ) [31], and the Insomnia Severity Index (ISI) [32]. Formal permission was obtained for the use of all three questionnaires.

#### 2.5.4. Dietary Quality

Dietary quality was assessed in the combined intervention group using the alternate Mediterranean Diet (aMED) score [33], with component cut-offs adapted from Fung et al. [34] and interpreted in accordance with the Mediterranean diet pyramid [35].

### 2.6. Ethical Considerations

The study was conducted in accordance with the Declaration of Helsinki. The study protocol was approved by the Ethics Committee of HCZC (protocol code: Class 072-30/24-01/003; Ref. No. 251-510-03-20-24-12; approved on 5 November 2024) and by the Ethics Committee of the School of Medicine, University of Zagreb (protocol code: Class 641-01/25-02/04; Ref. No. 251-59-10106-25-111/105; approved on 20 June 2025). All participants provided written informed consent before enrolment.

### 2.7. Blinding and Measures to Reduce Bias

Blinding of participants and intervention providers was not feasible because of the nature of the study interventions. To minimise bias, standardised counselling procedures were used by FPs before referral to the intervention programme. Participants completed the questionnaires at home; to minimise misunderstanding, they were provided with contact information for clarification if needed. Potential sources of self-report and recall bias, as well as variability in health literacy, motivation, and functional capacity, were considered when interpreting the findings.

### 2.8. Statistical Analysis

Sample size estimation was based on repeated-measures analysis of variance (ANOVA) for differences in quantitative variables before and after the intervention. Assuming 80% power, an effect size of f = 0.3, a two-sided type I error rate of α = 0.05, equal group sizes, and two time points, the required sample size was 96 participants (24 per group). To allow for potential drop-out, the planned sample size was increased to 120 participants (30 per group).

Statistical analyses were performed using Statistica version 14 (Cloud Software Group, San Ramon, CA, USA) [36] (licenced to the University of Zagreb School of Medicine) and the open-source software Jamovi (Version 2.5 (The Jamovi Project, Sydney, NSW, Australia)) [37]. Analyses were conducted per protocol on a complete-case dataset. Only participants with available baseline and follow-up measurements who attended at least 70% of exercise sessions were included in the analysis [38]. In the combined group, participants additionally had to attend at least three of the four dietitian consultations. Participants with missing follow-up data and/or not meeting these adherence criteria were excluded from the analysis.

Continuous variables are presented as mean ± standard deviation (SD) and/or as median (25th–75th percentile), as appropriate. Categorical variables are presented as n (%). Normality of distribution was assessed using the Shapiro–Wilk test. Baseline characteristics were compared across the three groups using one-way ANOVA for approximately normally distributed continuous variables or the Kruskal–Wallis test for skewed continuous and count variables, and Fisher’s exact test for categorical variables.

Changes from baseline to 3 months were analysed within groups using paired-samples t-tests or Wilcoxon signed-rank tests, as appropriate. Between-group comparisons of change scores were performed using one-way ANOVA or the Kruskal–Wallis test, depending on data distribution, with effect sizes reported where applicable. Correlations between continuous variables were assessed using Spearman’s correlation coefficients. All tests were two-sided. A more conservative significance threshold of *p* < 0.01 was prespecified in order to reduce the risk of type I error across multiple outcomes.

## 3. Results

**Figure 1:**
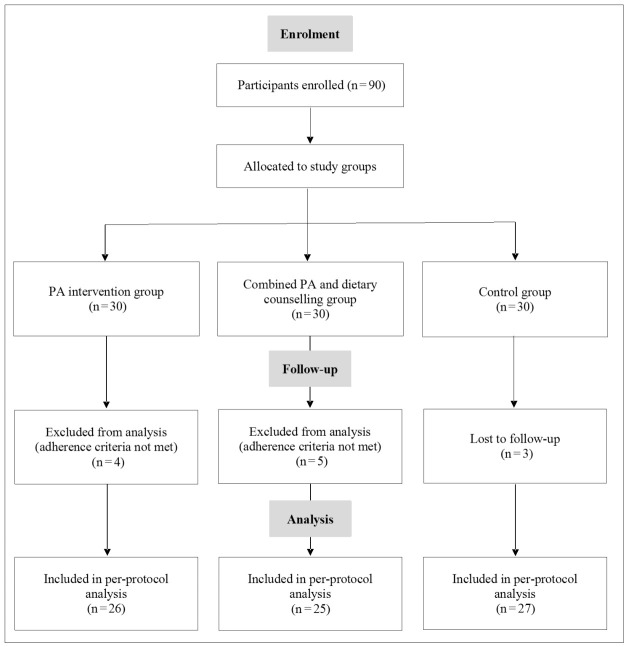
Flow diagram of participant enrolment, allocation, follow-up, and analysis (n = 90).

Of 90 enrolled participants, 78 (86.7%) completed the 3-month follow-up and were included in the per-protocol analyses (Figure 1). Exclusions were due to loss to follow-up (n = 3) or failure to meet prespecified adherence criteria (n = 9). The age distribution of participants who discontinued the programme was comparable to that of those who completed it.

No adverse events related to the intervention were reported.

Table S1 (Supplementary Materials) presents baseline and 3-month demographic and clinical characteristics by study group. Baseline characteristics were broadly comparable across groups. However, differences were observed for diastolic BP (*p* = 0.004) and number of medications per patient (*p* = 0.027).

Three-month follow-up values by study group are provided in Table S2 (Supplementary Materials). Changes from baseline to 3 months and between-group comparisons for clinical and laboratory outcomes are summarised in Table 1. Additional descriptive statistics for these outcomes are provided in Table S3 (Supplementary Materials).

Kidney-related outcomes (Table 1). The change in eGFR over 3 months did not differ significantly between groups (one-way ANOVA, *p* = 0.310). Mean (±SD) changes were 1.54 ± 9.39 mL/min/1.73 m^2^ in the PA group, 1.88 ± 6.55 mL/min/1.73 m^2^ in the combined group, and −0.78 ± 6.55 mL/min/1.73 m^2^ in the control group. Confidence intervals (CIs) were wide and overlapped across groups.

ACR showed a between-group *p* value of 0.017 (Kruskal–Wallis test, ε^2^ = 0.106), which did not meet the prespecified significance threshold (*p* < 0.01). Mean ± SD changes were −1.10 ± 6.37, −0.86 ± 2.88, and 1.18 ± 3.13, with corresponding 95% CIs of −3.68 to 1.47, −2.05 to 0.33, and −0.06 to 2.42, respectively.

Other outcomes (Table 1). A between-group difference was observed for FPG (*p* = 0.029, ε^2^ = 0.092), which did not meet the prespecified significance threshold. Systolic BP showed a borderline between-group difference (*p* = 0.010). Baseline diastolic BP differed between groups, limiting the interpretation of between-group comparisons of change.

Table 2 presents baseline and 3-month KDIGO G–A heat maps summarising participants’ risk categories by study group.

Baseline and 3-month values for SF-36, ISI, and IPAQ by study group are provided in Tables S4–S6 (Supplementary Materials).

Table 3 summarises changes in SF-36 domain and summary scores from baseline to 3 months by study group.

**Figure 2:**
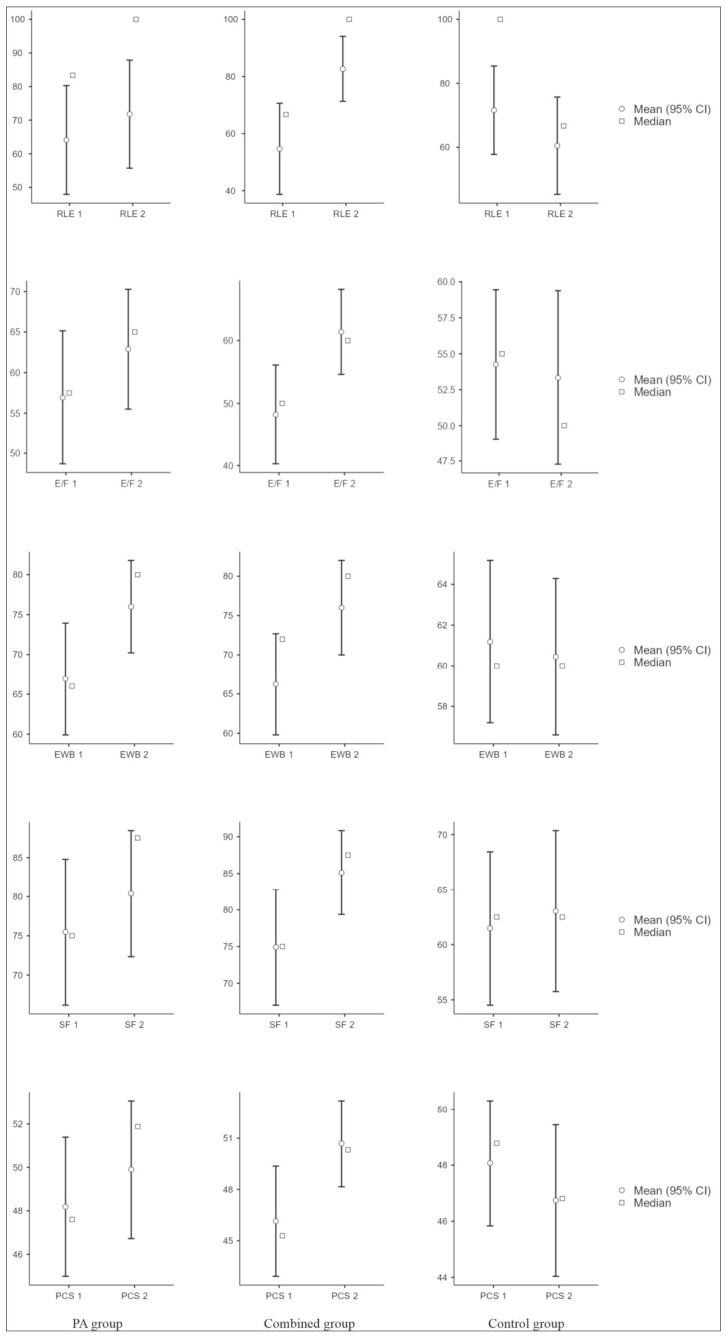
SF-36 domain and summary scores at baseline and 3 months by study group. PA: physical activity; RLE: role limitations due to emotional problems; E/F: energy/fatigue; EWB: emotional well-being; SF: social functioning; PCS: Physical Component Summary. Within-group change was assessed using the Wilcoxon signed-rank test.

Within-group improvements were observed for selected SF-36 domains in the intervention groups; however, interpretation focuses on between-group comparisons, given the susceptibility of within-group changes to expectancy and regression effects (Table 3). Between-group differences in change scores were observed for role limitations due to emotional problems (RLE), energy/fatigue (E/F), and emotional well-being (EWB) (Kruskal–Wallis test; all *p* < 0.001, ε^2^ ≈ 0.181–0.216), with larger median improvements in the intervention groups (Figure 2).

Table 4 presents changes in sleep-related outcomes assessed using the ISI by study group.

**Figure 3:**
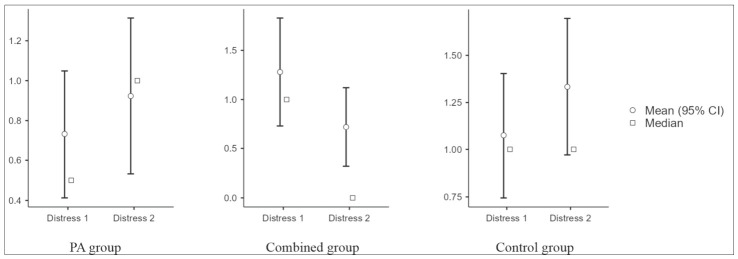
ISI distress domain scores at baseline and 3 months by study group. PA: physical activity. Within-group change was assessed using the Wilcoxon signed-rank test.

Change in the ISI total score did not differ between groups. A between-group difference was observed for the distress domain (Kruskal–Wallis test; *p* = 0.004; ε^2^ = 0.144), while no statistically significant differences were observed for the other domains (Figure 3). Sleep maintenance showed a between-group difference (*p* = 0.011, ε^2^ = 0.118) that did not meet the prespecified significance threshold.

Table 5 presents changes in IPAQ scores from baseline to 3 months by study group.

**Figure 4:**
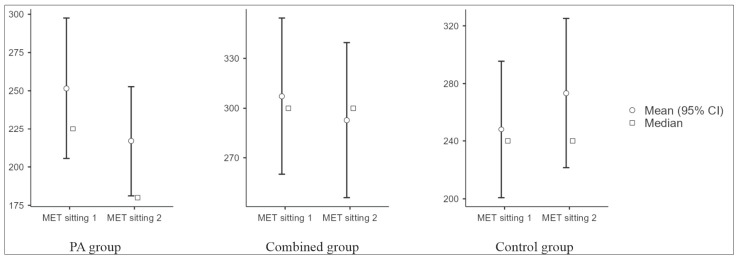
Daily sitting time at baseline and after 3 months by study group. PA: physical activity; MET: metabolic equivalent. Within-group change was assessed using the Wilcoxon signed-rank test.

**Figure 5:**
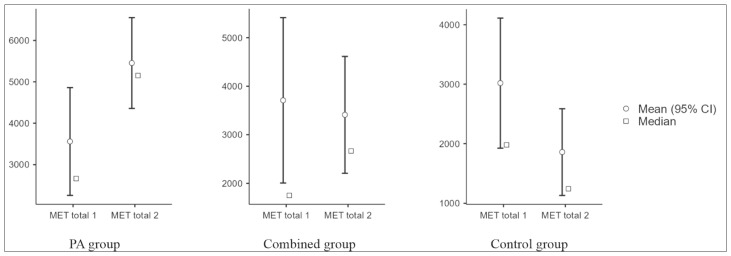
Total PA (MET-min/week) at baseline and after 3 months by study group. PA: physical activity; MET: metabolic equivalent. Within-group change was assessed using the Wilcoxon signed-rank test.

Sedentary time decreased in the PA (median −60 min/day) and the combined group (−30 min/day) and increased in the control group (+20 min/day), with a significant between-group difference (*p* = 0.006; ε^2^ = 0.133) (Figure 4). Total metabolic equivalent (MET) increased in the PA group (+1554 MET-min/week), changed little in the combined group (+60 MET-min/week), and decreased in controls (−240 MET-min/week), with a significant between-group difference (Kruskal–Wallis test; *p* < 0.001; ε^2^ = 0.225); the largest changes were observed in the PA group (Figure 5). Changes in PA and sedentary time were consistent with the supervised exercise intervention and were less pronounced in the combined group.

In the combined group (n = 25), the mean aMED score increased from 4.08 ± 1.75 at baseline to 4.92 ± 1.93 at 3 months. Median (25th–75th percentile) values were 5 (3.00–5.00) at baseline and 5 (3.00–6.00) at 3 months, with a significant within-group change (Wilcoxon signed-rank test, *p* = 0.002). Improvements were accompanied by higher component scores for several food groups, although overall changes in dietary quality were modest.

## 4. Discussion

### 4.1. Main Findings

In this short-term preliminary analysis, structured PA, with or without dietary counselling, was associated with improvements in several behavioural and patient-reported outcomes, particularly sedentary time, total PA, EWB, and E/F.

No statistically significant between-group effects were observed for the primary kidney-related outcomes, eGFR and ACR, over the 3-month follow-up. These findings should be interpreted cautiously in the context of the short intervention period, the relatively small sample, and the known biological variability of kidney-related measures in early-stage CKD.

### 4.2. Kidney-Related Outcomes

A clear short-term renal effect was not demonstrated in this study, which is not unexpected in early-stage CKD. In this phase of disease, changes in eGFR and albuminuria are often modest, biologically variable, and difficult to detect over short follow-up periods. Although mean eGFR increased in both intervention groups and decreased slightly in controls, and ACR changes numerically favoured the intervention groups, these findings remain exploratory. KDIGO risk stratification was also largely stable from baseline to 3 months, and any observed cell-to-cell shifts should be interpreted cautiously given the short follow-up and complete-case sample.

Our findings are consistent with recent evidence suggesting that lifestyle interventions in non-dialysis CKD may improve behavioural and patient-reported outcomes, while effects on kidney-related parameters remain modest or inconsistent, particularly in short-term studies [21,22,39,40]. This literature remains relatively limited in early-stage CKD, as many intervention studies have focused on advanced CKD or dialysis populations. Meta-analyses have reported small increases in eGFR (~2–3 mL/min/1.73 m^2^), but these estimates are often driven by small, short-term trials and may attenuate in longer studies [41,42]. Similarly, larger randomised trials have generally not shown a clear benefit on kidney function, although one study reported a greater reduction in ACR with one training modality compared with another [43]. Dietary interventions also appear to have less consistent effects on eGFR, but may produce greater improvements in albuminuria and systolic BP [22]. This is particularly relevant as nutritional guidance in CKD has increasingly shifted from single-nutrient targets towards overall dietary quality, although relatively few trials have evaluated whole dietary-pattern interventions [25,44,45].

### 4.3. Secondary Clinical, Behavioural, and Patient-Reported Outcomes

Although FPG showed a between-group difference, this did not meet the prespecified significance threshold and may reflect random variation rather than a robust intervention effect. BP findings should be interpreted cautiously, as between-group comparisons of change may partly reflect regression to the mean rather than a true intervention effect. No detectable changes were observed in medication counts, OTC/supplement use, smoking, or alcohol consumption, which is unsurprising given the short follow-up and the relative stability of these behaviours over 3 months.

Our analysis of HRQoL showed improvements concentrated in the intervention groups, particularly in the combined group, suggesting short-term benefits for emotional well-being and vitality, which is broadly consistent with recent evidence indicating that exercise interventions in non-dialysis CKD may improve mental and general HRQoL domains [42], while lifestyle interventions more broadly are more often associated with improvements in specific HRQoL domains than with uniform effects across all domains [22]. Within-group findings should be interpreted cautiously, as a minimal clinically important difference (MCID) of ~3–5 points is commonly used [46], and smaller changes may have limited clinical relevance.

Sleep-related outcomes showed a similar pattern. Greater improvement in the ISI distress domain in the combined group suggests a possible reduction in perceived sleep-related burden, whereas the finding for SM did not meet the prespecified significance threshold. This is compatible with recent evidence indicating that sleep and lifestyle behaviours are interrelated in CKD [47], although our findings remain exploratory.

Taken together, these results suggest that the intervention may have had a modest effect on subjective aspects of well-being and sleep, but these findings should be interpreted cautiously in the context of multiple comparisons.

Changes in PA behaviour were among the most clinically relevant findings of the study. Sedentary time decreased in both intervention groups, which is important given the global public health burden of sedentary lifestyles, particularly among multimorbid populations. Total PA increased most markedly in the PA group, suggesting that structured exercise may be more effective in increasing overall activity volume than in shifting specific intensity domains. In early-stage CKD, such behavioural changes may be important in their own right, as they align with the broader goals of integrated CKD care, including support for self-management, physical functioning, and longer-term risk-factor modification [48,49].

Improvements in selected aMED components in the combined group may reflect the practical and culturally acceptable nature of the counselling approach. However, the absence of change in some components, such as daily legume intake, also suggests that certain dietary habits may be more resistant to short-term modification and may require longer intervention and sustained support.

### 4.4. Implications for PHC and Integrated CKD Care

The direction of eGFR change, together with the observed improvements in PA and selected patient-reported outcomes, suggests that structured, supervised PA, alone or combined with dietitian-led dietary counselling, may have practical value in early-stage CKD even when a clear short-term renal effect is not demonstrable. This is clinically relevant because management of early-stage CKD in PHC depends not only on pharmacological treatment, but also on risk-factor modification, self-management support, and continuity of care, with FPs playing a central role in early identification, longitudinal monitoring, and coordination of lifestyle-oriented care [50,51].

This is consistent with contemporary CKD care models [12,13,14,15,16,52] and KDIGO [3] recommendations, which emphasise integrated, patient-centred, multidisciplinary care and communication between PHC and specialist teams [3]. The present intervention reflects part of this broader direction of CKD care, which increasingly relies on an interdisciplinary team and would ideally also include nephrology nurses contributing to education, care coordination, and self-management support [18,19], while dietitians, kinesiologists, and other trained professionals contribute to lifestyle counselling and related supportive care. Similarly, the growing literature on frailty in CKD [23,24] highlights the importance of preserving physical functioning and supporting early intervention before greater disability and care complexity develop.

Because this model is not routine in Croatian PHC, broader implementation would require clear protocols, defined team roles, adequate resources, and strategies to support adherence and raise clinician awareness of the importance of addressing PA and diet in CKD care [9,51,53]. This is particularly relevant because CKD-specific exercise recommendations remain incompletely standardised in everyday practice, and practical guidance is still underused [40,53]. The present findings may therefore be less important as proof of short-term renal efficacy than as evidence that structured lifestyle-oriented support can be delivered within PHC and aligned with the broader goals of integrated CKD care.

Although a 3-month intervention is feasible in PHC and broadly aligns with KDIGO 3–6-month reassessment intervals [3], longer intervention and follow-up with larger samples are needed to determine whether behavioural changes are sustained and whether they translate into measurable renal benefit over time. Future studies should include 6–12-month follow-up, consider telephone or hybrid follow-up approaches, and explore whether “booster” contacts improve maintenance. The planned doctoral dissertation, including a dietary-only group, should also help clarify the independent and additive effects of dietary counselling and PA.

### 4.5. Strengths and Limitations

A major strength of this study is its intervention model, which integrates supervised kinesiologist-led exercise and dietitian-led dietary counselling within a Croatian PHC setting. Group allocation and inclusion of a control group supported internal validity. The interdisciplinary approach involving FPs, a kinesiologist, and a dietitian supports comprehensive early CKD management and may enhance patient engagement and adherence. Validated Croatian-language instruments were used for behavioural and patient-reported outcomes. The prespecified *p* < 0.01 threshold reduced the risk of Type I error across multiple outcomes.

Several limitations should be acknowledged. The non-randomised design limits causal inference, and the modest sample size reduced power to detect smaller but potentially clinically meaningful differences, particularly for kidney-related outcomes. Although the 3-month follow-up broadly aligns with KDIGO recommendations to reassess key risk factors every 3–6 months, it was likely too short to detect reliable changes in eGFR, albuminuria, or KDIGO risk category in early-stage CKD; longer follow-up is needed to assess sustainability and potential renal and cardiometabolic effects. The absence of a formal screening log, together with the per-protocol complete-case approach and adherence-based exclusions, may have introduced selection bias and favoured more motivated participants. Questionnaires completed at home may have introduced misunderstanding, recall bias, or social desirability bias. Because creatinine-based eGFR estimates were used rather than measured GFR, exercise-related changes in muscle mass may have influenced kidney function estimates [40]. The aMED score does not fully capture some aspects of contemporary dietary patterns, including ultra-processed foods. Transferability should also be interpreted cautiously, as broader applicability to other PHC systems will depend on local resources, workforce availability, and the extent of multidisciplinary collaboration in routine practice. The predominantly older sample further limits applicability to younger adults with CKD. Accordingly, these findings are best interpreted as evidence of feasibility and short-term relevance within one PHC setting, rather than as proof of broadly generalisable effectiveness.

## 5. Conclusions

In adults with early-stage CKD managed in PHC, structured PA, with or without dietary counselling, improved PA behaviour and selected patient-reported outcomes, but did not demonstrate statistically significant short-term effects on kidney-related outcomes. These findings support the feasibility and potential clinical relevance of integrating lifestyle-oriented interventions into PHC as part of multidisciplinary CKD care. However, given the non-randomised design, modest sample size, and short follow-up, the results should be interpreted cautiously, and larger, longer-term studies are needed to clarify their renal effects and broader applicability.

## Figures and Tables

**Table 1:** Changes in measured parameters from baseline to 3 months by study group.

Outcome (Units)	Groups Comparable at Baseline	PA Group	Combined Group	Control Group	*p*
- sCr (µmol/L)	yes	−1.58 ± 12.3	−2.28 ± 8.03	0.59 ± 9.71	0.507 *
- eGFR (mL/min/1.73 m^2^)	yes	1.54 ± 9.39	1.88 ± 6.55	−0.78 ± 6.55	0.310 *
- ACR (mg/mmol)	yes	−1.10 ± 6.37	−0.86 ± 2.88	1.18 ± 3.13	0.017 **
- FPG (mmol/L)	yes	0.20 ± 0.85	−0.30 ± 0.76	0.79 ± 2.27	0.029 **
- HbA1c (%)	yes	0.05 ± 0.77	−0.14 ± 0.47	0.24 ± 0.54	0.118 **
- Cholesterol (mmol/L)	yes	−0.02 ± 0.91	−0.13 ± 0.78	0.03 ± 0.89	0.997 **
- LDL (mmol/L)	yes	0.09 ± 0.76	−0.06 ± 0.54	0.00 ± 0.76	0.965 **
- HDL (mmol/L)	yes	−0.057 ± 0.22	−0.01 ± 0.19	0.02 ± 0.19	0.652 **
- TG (mmol/L)	yes	−0.12 ± 0.56	0.04 ± 0.37	0.04 ± 0.71	0.432 **
Body mass index, kg/m^2^	yes	−0.19 ± 0.85	−0.23 ± 0.83	0.11 ± 0.31	0.071 **
Chronic diagnoses per patient	yes	−0.15 ± 0.97	−0.32 ± 0.99	0.33 ± 0.62	0.025 **
Medications per patient	no	−0.19 ± 1.02	0.04 ± 0.98	0.56 ± 1.22	0.017 **
OTC/supplements per patient	yes	−0.039 ± 0.77	−0.4 ± 0.91	0.07 ± 0.39	0.077 **
Blood pressure, mmHg	yes	−9.19 ± 16.6	−6.60 ± 15.9	1.96 ± 6.82	0.010 **
- Systolic - Diastolic	no	−5.88 ± 11.7	−4.16 ± 12.7	2.22 ± 6.81	0.077 **

PA: physical activity; sCr: serum creatinine; eGFR: estimated glomerular filtration rate; ACR: albumin-to-creatinine ratio; FPG: fasting plasma glucose; HbA1c: haemoglobin A1c; LDL: low-density lipoprotein; HDL: high-density lipoprotein; TG: triglycerides; OTC: over-the-counter. Values are presented as mean ± standard deviation (SD). *p* values refer to between-group comparisons of 3-month changes and were calculated using one-way ANOVA (*) or the Kruskal–Wallis test (**).

**Table 2:** KDIGO eGFR–albuminuria (G–A) heat maps at baseline and 3-month follow-up.

(A) baseline									
KDIGO CKD Risk Category	A1			A2			A3		
G1	0	0	0	4 (15.38)	3 (12.00)	0	0	3 (11.11)	0
G2	4 (15.38)	3 (12.00)	1 (3.70)	4 (15.38)	6 (24.00)	0	0	7 (25.93)	0
G3a	11 (42.31)	11 (44.00)	11 (40.74)	0	0	1 (4.00)	1 (3.85)	0	0
G3b	2 (7.69)	0	3 (11.11)	0	1 (4.00)	0	0	0	0
(B) three-month follow-up									
KDIGO CKD risk category	A1			A2			A3		
G1	1 (3.85)	0	0	2 (7.69)	1 (4.00)	4 (14.81)	0	0	0
G2	7 (26.92)	7 (28.00)	2 (7.40)	5 (19.23)	5 (20.00)	5 (18.52)	0	0	0
G3a	8 (30.77)	10 (40.00)	11 (40.74)	0	0	3 (11.11)	0	1 (4.00)	0
G3b	2 (7.69)	0	1 (3.70)	1 (3.85)	1 (4.00)	1 (3.70)	0	0	0

KDIGO: Kidney Disease: Improving Global Outcomes; CKD: chronic kidney disease; eGFR: estimated glomerular filtration rate; ACR: albumin-to-creatinine ratio; G–A: glomerular filtration rate and albuminuria classification; G1–G3b: eGFR categories; A1–A3: albuminuria categories; PA: physical activity. Rows indicate eGFR categories (G1–G3b), and columns indicate albuminuria categories (A1–A3) based on the urinary ACR. Each cell displays the number of participants in the corresponding G–A category as n (%), by study group: PA group (n = 26), combined group (n = 25), and control group (n = 27). Cell colours reflect the KDIGO risk classification (green = low risk, yellow = moderately increased risk, orange = high risk, light red = very high risk, deep red = extremely high risk). Percentages are within each study group. Both heat maps include only participants with complete data.

**Table 3:** Changes in SF-36 domain and summary scores from baseline to 3 months by study group.

SF-36 Questionnaire Domain	PA Group	Combined Group	Control Group	*p*
PF Δ	2.5 (−15.0–18.8)	10.0 (−5.0–20.0)	0.0 (−7.5–5.0)	0.199
RLP Δ	0.0 (−18.8–0.0)	0.0 (0.0–25.0)	0.0 (−12.5–0.0)	0.814
RLE Δ	0.0 (0.0–25.0)	33.3 (0.0–66.7)	0.0 (−33.3–0)	<0.001
E/F Δ	5.0 (−3.75–10.0)	10.0 (5.0–20.0)	0.0 (−5.0–5.0)	<0.001
EWB Δ	8.0 (0.0–12.0)	8.0 (0.0–20.0)	−4.0 (−4.0–2.0)	<0.001
SF Δ	0.0 (0.0–12.5)	12.5 (0.0–25.0)	0.0 (0.0–2.5)	0.082
P Δ	0.0 (0.0–10.0)	0.0 (−2.5–12.5)	0.0 (−7.5–1.25)	0.419
GH Δ	5.5 (−3.75–10.8)	5.0 (0–10.0)	−2.0 (−5–3.5)	0.036
Summary scores				
PCS Δ	0.7 (−2.9–4.8)	2.5 (−0.9–11.1)	−0.6 (−3.9–1.2)	0.019
MCS Δ	1.6 (−5.8–4.1)	1.8 (−2.3–4.8)	0.9 (−1.4–2.4)	0.609

Δ: change from baseline to 3 months (3-month value minus baseline value); SF-36: Short Form-36; PA: physical activity; PF: physical functioning; RLP: role limitations due to physical health; RLE: role limitations due to emotional problems; E/F: energy/fatigue; EWB: emotional well-being; SF: social functioning; P: pain; GH: general health; PCS: Physical Component Summary; MCS: Mental Component Summary. Values are presented as median (25th–75th percentile). *p* values in the last column refer to between-group comparisons of 3-month change scores (Kruskal–Wallis test).

**Table 4:** Changes in ISI questionnaire domains from baseline to 3 months by study group.

ISI Questionnaire Domain	PA Group	Combined Group	Control Group	*p*
SO Δ	0.00 (−0.75–0.00)	0.00 (0.00–0.00)	0.00 (0.00–0.00)	0.379
SM Δ	0.00 (0.00–0.00)	0.00 (−1.00–0.00)	0.00 (0.00–1.00)	0.011
EMA Δ	0.00 (−0.75–0.00)	0.00 (0.00–0.00)	0.00 (−0.50–0.50)	0.809
SS Δ	0.00 (−1.00–0.00)	0.00 (−1.00–0.00)	0.00 (−1.00–0.00)	0.922
DI Δ	0.00 (−1.00–0.00)	0.00 (−1.00–0.00)	0.00 (0.00–0.00)	0.153
N Δ	0.00 (0.00–0.00)	0.00 (−1.00–0.00)	0.00 (0.00–0.00)	0.497
D Δ	0.00 (0.00–0.00)	−1.00 (−1.00–0.00)	0.00 (0.00–1.00)	0.004
ISI total Δ	0.00 (−3.75–0.00)	0.00 (−3.00–0.00)	0.00 (0.00–1.00)	0.102

Δ: change from baseline to 3 months (3-month value minus baseline value); ISI: Insomnia Severity Index; PA: physical activity; SO: sleep onset; SM: sleep maintenance; EMA: early morning awakening; SS: sleep satisfaction; DI: daytime impairment; N: noticeability; D: distress. Values are presented as median (25th–75th percentile). *p* values in the last column refer to between-group comparisons of 3-month change scores (Kruskal–Wallis test).

**Table 5:** Changes in IPAQ domain scores from baseline to 3 months by study group.

IPAQ Domain	PA Group	Combined Group	Control Group	*p*
MET vigorous Δ	0 (0.0–1380)	0 (0.0–800)	0 (0.0–0.0)	0.021
MET moderate Δ	270 (−390–930)	60 (−600–480)	−200 (−510–0.0)	0.015
MET walking Δ	0 (−297–965)	0 (−330–495)	0 (−396–57.8)	0.463
Sitting time Δ	−60 (−120–0.0)	−30 (−60.0–0.0)	20 (0.0–60.0)	0.006
MET total Δ	1554 (364–2750)	60 (−1062–1242)	−240 (−612–0.0)	<0.001

Δ: change from baseline to 3 months (3-month value minus baseline value); IPAQ: International Physical Activity Questionnaire; PA: physical activity; MET: metabolic equivalent. Values are presented as median (25th–75th percentile). *p* values in the last column refer to between-group comparisons of 3-month change scores (Kruskal–Wallis test).

## Data Availability

The original contributions presented in this study are included in the article/Supplementary Materials. Further inquiries can be directed to the corresponding author.

## Supplementary Materials

The following supporting information can be downloaded at: https://www.mdpi.com/article/10.3390/jcm15083169/s1, Table S1: Baseline demographic and clinical characteristics by study group; Table S2: Three-month follow-up demographic and clinical characteristics by study group; Table S3: Additional descriptive statistics for 3-month changes in outcomes presented in Table 1; Tables S4–S6: Baseline and 3-month values by study group for SF-36, ISI, and IPAQ.

## Abbreviations

ACR	albumin-to-creatinine ratio
aMED	alternate Mediterranean Diet
ANOVA	analysis of variance
BMI	body mass index
BP	blood pressure
CKD	chronic kidney disease
CKD-EPI	Chronic Kidney Disease Epidemiology Collaboration
CIs	confidence intervals
D	distress
DI	daytime impairment
E/F	energy/fatigue
eGFR	estimated glomerular filtration rate
EMA	early morning awakening
EWB	emotional well-being
FPs	family physicians
FMPs	family medicine practices
FPG	fasting plasma glucose
G–A	glomerular filtration rate and albuminuria classification
GFR	glomerular filtration rate
GH	general health
HbA1c	glycated haemoglobin
HCZC	Health Centre Zagreb—Centar
HDL	high-density lipoprotein
HRQoL	health-related quality of life
IPAQ	International Physical Activity Questionnaire
ISI	Insomnia Severity Index
KDIGO	Kidney Disease: Improving Global Outcomes
KDOQI	Kidney Disease Outcomes Quality Initiative
LDL	low-density lipoprotein
MCID	minimal clinically important difference
MCS	Mental Component Summary
MET	metabolic equivalent
N	noticeability
OTC	over-the-counter
P	pain
PA	physical activity
PCS	Physical Component Summary
PF	physical functioning
PHC	primary healthcare
RLE	role limitations due to emotional problems
RLP	role limitations due to physical health
sCr	serum creatinine
SD	standard deviation
SF	social functioning
SF-36	Short Form-36
SM	sleep maintenance
SO	sleep onset
SS	sleep satisfaction
TG	triglycerides
TIDieR	Template for Intervention Description and Replication
TREND	Transparent Reporting of Evaluations with Nonrandomised Designs

## References

[1] Bello A.K., Okpechi I.G., Levin A., Ye F., Saad S., Zaidi D., Jha V., Malik C., Osman M.A., Tonelli M. (2023). ISN–Global Kidney Health Atlas: A Report by the International Society of Nephrology: An Assessment of Global Kidney Health Care Status Focussing on Capacity, Availability, Accessibility, Affordability and Outcomes of Kidney Disease.

[2] Croatian Institute of Public Health Svjetski dan Bubrega, 14. Ožujka 2024. [World Kidney Day, 14 March 2024]. https://www.hzjz.hr/aktualnosti/svjetski-dan-bubrega-14-ozujka-2024/.

[3] (2024). Kidney Disease: Improving Global Outcomes (KDIGO) CKD Work Group. KDIGO 2024 Clinical Practice Guideline for the Evaluation and Management of Chronic Kidney Disease. Kidney Int..

[4] GBD 2021 Forecasting Collaborators (2024). Burden of disease scenarios for 204 countries and territories, 2022–2050: A forecasting analysis for the Global Burden of Disease Study 2021. Lancet.

[5] Flaherty C.M., Surapaneni A., Seegmiller J.C., Coresh J., Grams M.E., Ballew S.H. (2024). CKD prevalence and incidence in older adults using estimated GFR with different filtration markers: The Atherosclerosis Risk in Communities Study. Kidney Med..

[6] Beridze G., Dai L., Carrero J.J., Marengoni A., Vetrano D.L., Calderón-Larrañaga A. (2025). Associations between multimorbidity and kidney function decline in old age: A population-based cohort study. J. Am. Geriatr. Soc..

[7] Baker L.A., March D.S., Wilkinson T.J., Billany R.E., Bishop N.C., Castle E.M., Chilcot J., Davies M.D., Graham-Brown M.P.M., Greenwood S.A. (2022). Clinical practice guideline exercise and lifestyle in chronic kidney disease. BMC Nephrol..

[8] Rampersad C., Brar R., Connelly K., Komenda P., Rigatto C., Prasad B., Bohm C., Tangri N. (2021). Association of physical activity and poor health outcomes in patients with advanced CKD. Am. J. Kidney Dis..

[9] Battaglia Y., Baciga F., Bulighin F., Amicone M., Mosconi G., Storari A., Brugnano R., Pozzato M., Motta D., D’aLessandro C. (2024). Physical activity and exercise in chronic kidney disease: Consensus statements from the Physical Exercise Working Group of the Italian Society of Nephrology. J. Nephrol..

[10] Levin A., Okpechi I.G., Caskey F.J., Yang C.W., Tonelli M., Jha V. (2023). Perspectives on early detection of chronic kidney disease: The facts, the questions, and a proposed framework for 2023 and beyond. Kidney Int..

[11] Nagib S.N., Abdelwahab S., Amin G.E.E., Allam M.F. (2021). Screening and early detection of chronic kidney disease at primary healthcare. Clin. Exp. Hypertens..

[12] Burton J.O., Frankel A.H., Kwon K., Marqués M., Jiang G., Wang J., McCafferty K. (2026). Transformative global models for CKD care: Case studies and strategies. Clin. Kidney J..

[13] Annadanam S., Garg G., Fagerlin A., Powell C., Chen E., Segal J.H., Ojo A., Nunes J.W. (2023). Patient-centered outcomes with a multidisciplinary CKD care team approach: An observational study. Kidney Med..

[14] Shimonov D., Tummalapalli S.L., Donahue S., Narayana V., Wu S., Walters L.S., Billman R., Desiderio B., Pressman S., Fielding O. (2024). Clinical outcomes of a novel multidisciplinary care program in advanced kidney disease (PEAK). Kidney Int. Rep..

[15] Nkunu V., Wiebe N., Bello A., Campbell S., Tannor E., Varghese C., Stanifer J., Tonelli M. (2022). Update on existing care models for chronic kidney disease in low- and middle-income countries: A systematic review. Can. J. Kidney Health Dis..

[16] Górriz J.L., Alcázar Arroyo R., Arribas P., Artola S., Cinza-Sanjurjo S., de la Espriella R., Escalada J., García-Matarín L., Martínez L., Julián J.C. (2024). Multidisciplinary Delphi consensus on challenges and key factors for an optimal care model in chronic kidney disease. Nefrologia.

[17] Attalla M., Friedman Z., McKeown S., Harel Z., Hingwala J., Molnar A.O., Norman P., Silver S.A. (2020). Characteristics and effectiveness of dedicated care programs for patients starting dialysis: A systematic review. Kidney360.

[18] Andreoli D., Morales Palomares S., Mancin S., Parozzi M., Gazineo D., Palmisano A., Angileri S., Ricco M., Anastasi G., Savini S. (2025). Exploring the competencies of nephrology nurses: A comprehensive scoping review. Int. Nurs. Rev..

[19] Arooj H., Aman M., Hashmi M.U., Nasir Z., Zahid M., Abbas J., Amjad N., Maryam S., Farhan K. (2025). The impact of nurse-led care in chronic kidney disease management: A systematic review and meta-analysis. BMC Nurs..

[20] Villanego F., Naranjo J., Vigara L.A., Cazorla J.M., Montero M.E., García T., Torrado J., Mazuecos A. (2020). Impact of physical exercise in patients with chronic kidney disease: Systematic review and meta-analysis. Nefrologia.

[21] Tsuchida Y., Kabasawa H., Uno C., Nishioka S., Sofue T., Fujii N., Saitoh M., Narita I., Yamagata K., Hoshino J. (2025). Efficacy of combined exercise and nutritional intervention for nondialysis chronic kidney disease: A systematic review. J. Ren. Nutr..

[22] Neale E.P., Rosario V.D., Probst Y., Beck E., Tran T.B., Lambert K. (2023). Lifestyle interventions, kidney disease progression, and quality of life: A systematic review and meta-analysis. Kidney Med..

[23] Kennard A.L., Glasgow N.J., Rainsford S.E., Talaulikar G.S. (2024). Narrative review: Clinical implications and assessment of frailty in patients with advanced CKD. Kidney Int. Rep..

[24] Puri A., Lloyd A.M., Bello A.K., Tonelli M., Campbell S.M., Tennankore K., Davison S.N., Thompson S. (2025). Frailty assessment tools in chronic kidney disease: A systematic review and meta-analysis. Kidney Med..

[25] Ikizler T.A., Burrowes J.D., Byham-Gray L.D., Campbell K.L., Carrero J.J., Chan W., Fouque D., Friedman A.N., Ghaddar S., Goldstein-Fuchs D.J. (2020). KDOQI clinical practice guideline for nutrition in CKD: 2020 update. Am. J. Kidney Dis..

[26] Safdar F., Aslam A. (2025). Chronic kidney disease in the primary care setting: A narrative review. J. Gen. Fam. Med..

[27] IBM Corp (2017). IBM SPSS Statistics for Windows, Version 25.0.

[28] World Health Organization (2020). WHO Guidelines on Physical Activity and Sedentary Behaviour.

[29] Bailey R.L., Fulgoni V.L., Cowan A.E., Gaine P.C. (2021). Overview of dietary assessment methods for measuring intakes of foods, beverages, and dietary supplements in research studies. Curr. Dev. Nutr..

[30] Maslić Seršić D., Vuletić G. (2006). Psychometric evaluation and establishing norms of Croatian SF-36 health survey: Framework for subjective health research. Croat. Med. J..

[31] Ajman H., Đapić Štriga S., Novak D. (2015). Reliability of the Croatian short version of the International Physical Activity Questionnaire. Hrvat. Športskomed. Vjesn..

[32] Bastien C.H., Vallières A., Morin C.M. (2001). Validation of the Insomnia Severity Index as an outcome measure for insomnia research. Sleep Med..

[33] Hu E.A., Coresh J., Anderson C.A.M., Appel L.J., Grams M.E., Crews D.C., Mills K.T., He J., Scialla J., Rahman M. (2021). Adherence to healthy dietary patterns and risk of CKD progression and all-cause mortality: Findings from the CRIC (Chronic Renal Insufficiency Cohort) study. Am. J. Kidney Dis..

[34] Fung T.T., McCullough M.L., Newby P.K., Manson J.E., Meigs J.B., Rifai N., Willett W.C., Hu F.B. (2005). Diet-quality scores and plasma concentrations of markers of inflammation and endothelial dysfunction. Am. J. Clin. Nutr..

[35] Sofi F., Martini D., Angelino D., Cairella G., Campanozzi A., Danesi F., Dinu M., Erba D., Iacoviello L., Pellegrini N. (2025). Mediterranean diet: Why a new pyramid? An updated representation of the traditional Mediterranean diet by the Italian Society of Human Nutrition (SINU). Nutr. Metab. Cardiovasc. Dis..

[36] Cloud Software Group (2023). TIBCO Data Science–Workbench (Statistica), Version 14.1.0.

[37] The Jamovi Project (2024). Jamovi, Version 2.5.

[38] Teresi J.A., Yu X., Stewart A.L., Hays R.D. (2022). Guidelines for designing and evaluating feasibility pilot studies. Med. Care.

[39] Ikizler T.A., Robinson-Cohen C., Ellis C., Headley S.A.E., Tuttle K., Wood R.J., Evans E.E., Milch C.M., Moody K.A., Germain M. (2018). Metabolic effects of diet and exercise in patients with moderate to severe CKD: A randomized clinical trial. J. Am. Soc. Nephrol..

[40] Clyne N., Anding-Rost K. (2021). Exercise training in chronic kidney disease-effects, expectations and adherence. Clin. Kidney J..

[41] Zhang L., Wang Y., Xiong L., Luo Y., Huang Z., Yi B. (2019). Exercise therapy improves eGFR, and reduces blood pressure and BMI in non-dialysis CKD patients: Evidence from a meta-analysis. BMC Nephrol..

[42] Traise A., Dieberg G., Pearson M.J., Smart N.A. (2024). The effect of exercise training in people with pre-dialysis chronic kidney disease: A systematic review with meta-analysis. J. Nephrol..

[43] Hellberg M., Höglund P., Svensson P., Clyne N. (2019). Randomized controlled trial of exercise in CKD—The RENEXC study. Kidney Int. Rep..

[44] Shi H., Su X., Li C., Guo W., Wang L. (2022). Effect of a low-salt diet on chronic kidney disease outcomes: A systematic review and meta-analysis. BMJ Open.

[45] Ellis T., Kwon A.J., Hong M.Y. (2024). The effectiveness of telehealth intervention on chronic kidney disease management in adults: A systematic review. Mayo Clin. Proc. Digit. Health.

[46] Ware J.E., Kosinski M., Gandek B. (2000). SF-36 Health Survey: Manual & Interpretation Guide.

[47] Lin X., Lv J., Zhang S., Ma X., Zhang X., Wang C., Zhang T. (2024). Healthy lifestyle behaviors attenuate the effect of poor sleep patterns on chronic kidney disease risk: A prospective study from the UK Biobank. Nutrients.

[48] Evén G., Stenfors T., Jacobson S.H., Jernberg T., Franzén-Dahlin Å., Jäghult S., Kahan T., Spaak J. (2024). Integrated, person-centred care for patients with complex cardiovascular disease, diabetes mellitus and chronic kidney disease: A randomized trial. Clin. Kidney J..

[49] Ortiz A., Arreola Guerra J.M., Chan J.C.N., Jha V., Kramer H., Nicholas S.B., Pavkov M.E., Wanner C., Wong L.P., Cheung M. (2025). Preventing chronic kidney disease and maintaining kidney health: Conclusions from a Kidney Disease: Improving Global Outcomes (KDIGO) controversies conference. Kidney Int..

[50] Kushner P., Khunti K., Cebrián A., Deed G. (2024). Early identification and management of chronic kidney disease: A narrative review of the crucial role of primary care practitioners. Adv. Ther..

[51] Thornton J., Nagpal T., Reilly K., Stewart M., Petrella R. (2022). The ‘miracle cure’: How do primary care physicians prescribe physical activity with the aim of improving clinical outcomes of chronic disease? A scoping review. BMJ Open Sport Exerc. Med..

[52] Duru O.K., Alicic R., Vaduganathan M., Peter W.L.S., Roberts G.V., Rangaswami J., Nicholas S.B., Neumiller J.J., Mathew R.O., Gee P. (2025). A systematic literature review of coordinated care in cardiovascular-kidney-metabolic conditions. Mayo Clin. Proc. Innov. Qual. Outcomes.

[53] Wattanapisit A., Wattanapisit S., Wongsiri S. (2021). Overview of physical activity counseling in primary care. Korean J. Fam. Med..

