# Supplementary material for: Short-Term Effects of Structured Physical Activity With or Without Dietary Counselling in Early-Stage Chronic Kidney Disease Managed in Primary Care: A Non-Randomised Controlled Study
Source: J Clin Med. 2026 Apr 21;15(8):3169. doi: 10.3390/jcm15083169 (PMC13117030; doi:10.3390/jcm15083169)
Supplement: Supplementary file 1 [file jcm-15-03169-s001.zip › Table S1.pdf]

Table S1. Baseline demographic and clinical characteristics by study group.

PARTICIPANT CHARACTERISTICS	PA GROUP	COMBINED GROUP	CONTROL GROUP	p
Participants, number	26	25	27	
Age, Mean ± SD	69.8 ± 4.50	68.0 ± 4.68	69.6 ± 5.68	0.163*
Median (range)	70.5 (57−75)	69.0 (59−75)	70.0 (57−75)	
Sex, n (%)				0.204**
- Female	19 (73.08)	22 (88.00)	18 (66.67)	
- Male	7 (26.92)	3 (12.00)	9 (33.33)	
Body mass index, kg/m²	29.6 ± 5.26	30.6 ± 4.77	28.8 ± 4.76	0.346*
Obese, n (%)	8 (30.77)	14 (56.00)	8 (29.63)	
Overweight, n (%)	14 (53.85)	7 (28.00)	15 (55.56)	
Smokers, n (%)				0.971**
- Active	7 (26.92)	6 (24.00)	7 (25.93)	
- Former	2 (7.69)	1 (4.00)	3 (11.11)	
Pack-year per smoker	29.5 ± 17.4	22.5 ± 18.2	29.3 ± 20.4	0.965*
Alcohol consumers, n (%)				0.808**
- abstainers	15 (57.69)	17 (68.00)	17 (62.96)	
- low-risk	10 (38.46)	6 (24.00)	9 (33.33)	
- high-risk	1 (3.85)	2 (8.00)	1 (3.70)	
Chronic diagnoses per patient	4 (3.00−6.75)	4 (4.00−8.00)	6 (4.00−7.00)	0.283*
Medications per patient	3 (2.25−6.00)	4 (3.00−7.00)	6 (4.00−7.00)	0.027*
OTC/ supplements per patient	1 (0.00−2.00)	1 (0.00−3.00)	0 (0.00−1.00)	0.251*
Blood pressure, mmHg				
- Systolic	143 ± 13.6	144 ± 14.9	137 ± 12.5	0.625***
- Diastolic	88.9 ± 10.5	89.8 ± 10.2	81.5 ± 8.37	0.004***

PARTICIPANT CHARACTERISTICS	PA GROUP	COMBINED GROUP	CONTROL GROUP	p
Metabolic panel, mmol/L (FPG); % (HbA1c)				
- FPG	5.80 (5.23−6.42)	6.20 (5.60−7.10)	5.80 (5.20−6.95)	0.346*
- HbA1c, n (%)	13 (50.00)	14 (56.00)	16 (59.26)	0.953*
Median (25th–75th percentile)	6.40 (5.80−7.00)	6.30 (5.82−6.88)	6.45 (5.88−7.12)	
Lipid profile, mmol/L				
- Cholesterol	4.60 (3.65−5.90)	4.90 (3.60−5.80)	4.70 (3.65−5.90)	0.896*
- LDL	2.35 (1.83−3.70)	2.60 (1.80−3.80)	2.70 (1.75−3.55)	0.907*
- HDL	1.40 (1.30−1.60)	1.40 (1.20−1.60)	1.42 (1.15−1.75)	0.850***
- TG	1.35 (1.02−1.84)	1.10 (0.90−1.70)	1.50 (1.20−2.69)	0.058*
Renal panel, µmol/L (sCr); mL/min/1.73 m² (eGFR); mg/mmol (ACR)				
- sCr	92.5 (80.5−116)	93 (75.0−106)	96 (75.7−119)	0.652*
- eGFR	58.0 (54.0−69.0)	59.0 (53.0−77.0)	59 (52.0−79.5)	0.931*
- ACR	0.53 (0.13−3.55)	1.40 (0.40−3.70)	1.40 (0.10−3.05)	0.662*

PA: physical activity; OTC: over-the-counter; FPG: fasting plasma glucose; HbA1c: haemoglobin A1c; LDL: low-density lipoprotein; HDL: high-density lipoprotein; TG: triglycerides; sCr: serum creatinine; eGFR: estimated glomerular filtration rate; ACR: albumin-to-creatinine ratio. Values are presented as n (%), mean ± standard deviation (SD), and median (range or 25th–75th percentile), as appropriate. Count variables (number of diagnoses, medications, and OTC/supplements) and laboratory parameters are reported as median (25th–75th percentile) to provide robust estimates in this relatively small sample. Between-group baseline p values are shown in a separate column and were calculated using the Kruskal–Wallis test (*), Fisher’s exact test (**), or one-way ANOVA (***).
