# Supplementary material for: Short-Term Effects of Structured Physical Activity With or Without Dietary Counselling in Early-Stage Chronic Kidney Disease Managed in Primary Care: A Non-Randomised Controlled Study
Source: J Clin Med. 2026 Apr 21;15(8):3169. doi: 10.3390/jcm15083169 (PMC13117030; doi:10.3390/jcm15083169)
Supplement: Supplementary file 1 [file jcm-15-03169-s001.zip › Table S2.pdf]

Table S2. Three-month follow-up demographic and clinical characteristics by study group.

PARTICIPANT CHARACTERISTICS	PA GROUP		COMBINED GROUP		CONTROL GROUP	
	3-month	p	3-month	p	3-month	p
Participants, number	26		25		27	
Age, Mean ± SD	69.8 ± 4.50		68.0 ± 4.68		69.6 ± 5.68	
Median (range)	70.5 (57−75)		69.0 (59−75)		70.0 (57−75)	
Sex, n (%)<br>- Female	19 (73.08)		22 (88.00)		18 (66.67)	
- Male	7 (26.92)		3 (12.00)		9 (33.33)	
Body mass index, kg/m²	29.4 ± 4.94	0.252*	30.4 ± 4.56	0.180*	28.9 ± 4.87	0.097**
Obese, n (%)	8 (30.77)		14 (56.00)		8 (29.63)	
Overweight, n (%)	14 (53.85)		7 (28.00)		15 (55.56)	
Smokers, n (%)<br>- Active	7 (26.92)		6 (24.00)		7 (25.93)	
- Former	2 (7.69)		1 (4.00)		3 (11.11)	
Pack-year per smoker	26.3 ± 17.0		22.5 ± 18.2		29.3 ± 20.4	
Alcohol consumers, n (%)<br>- abstainers	15 (57.69)		15 (60.00)		18 (66.67)	
- low-risk	10 (38.46)		8 (32.00)		8 (29.63)	
- high-risk	1 (3.85)		2 (8.00)		1 (3.79)	
Chronic diagnoses per patient	4 (3.00−6.00)		4 (3.00−7.00)		6 (4.50−7.00)	
Medications per patient	3 (2.00−5.75)		4 (2.00−7.00)		6 (5.00−7.00)	
OTC/ supplements per patient	1 (0.00−1.75)		0 (0.00−2.00)		1 (0.00−1.00)	
Blood pressure, mmHg<br>- Systolic	134 ± 18.3	0.009*	138 ± 19.8	0.063**	139 ± 11.9	0.147*
- Diastolic	82.9 ± 9.99	0.017**	85.6 ± 9.37	0.508**	83.7 ± 9.00	0.102*

PARTICIPANT CHARACTERISTICS	PA GROUP		COMBINED GROUP		CONTROL GROUP	
	3-month	p	3-month	p	3-month	p
Metabolic panel, mmol/L (FPG); % (HbA1c)						
- FPG	5.80 (5.23−6.97)	0.255**	5.80 (5.40−6.80)	0.107**	6.10 (5.45−7.50)	0.057**
- HbA1c, n (%)	13 (50.00)		14 (56.00)		16 (59.26)	
Median (25th–75th percentile)	6.20 (6.10−7.40)	0.599**	6.40 (5.88−6.63)	0.307**	6.50 (5.80−7.30)	0.081**
Lipid profile, mmol/L						
- Cholesterol	4.70 (3.50−5.38)	0.915*	4.40 (3.60−5.80)	0.422*	4.80 (3.80−6.00)	0.896**
- LDL	2.80 (1.83−3.27)	0.542*	2.50 (1.80−3.50)	0.611*	2.77 (2.00−3.45)	0.727**
- HDL	1.40 (1.13−1.60)	0.192*	1.40 (1.20−1.60)	0.765*	1.40 (1.15−1.77)	0.634**
- TG	1.20 (1.02−1.60)	0.283*	1.10 (1.00−1.60)	0.598*	1.70 (1.15−2.45)	0.875**
Renal panel, µmol/L (sCr); mL/min/1.73 m² (eGFR); mg/mmol (ACR)						
- sCr	90.5 (75.5−114)	0.519*	91 (72.0−97.0)	0.169*	99 (75.0−119)	0.754*
- eGFR	62.0 (54.3−73.0)	0.412*	60 (56.0−78.0)	0.154*	57 (50.0−77.5)	0.543*
- ACR	0.55 (0.13−3.00)	0.256**	1.00 (0.30−3.20)	0.098**	2.20 (0.35−4.60)	0.036**

PA: physical activity; OTC: over-the-counter; FPG: fasting plasma glucose; HbA1c: haemoglobin A1c; LDL: low-density lipoprotein; HDL: high-density lipoprotein; TG: triglycerides; sCr: serum creatinine; eGFR: estimated glomerular filtration rate; ACR: albumin-to-creatinine ratio. Values are presented as n (%), mean ± standard deviation (SD), and median (range or 25th–75th percentile), as appropriate. Count variables (number of diagnoses, medications, and OTC/supplements) and laboratory parameters are reported as median (25th–75th percentile) to provide robust estimates in this relatively small sample. Within-group p values compare baseline and 3-month follow-up values within each group and

were calculated using the paired-samples t-test (*) or Wilcoxon signed-rank test (**). Baseline values are reported in Table S1.
