# Supplementary material for: Short-Term Effects of Structured Physical Activity With or Without Dietary Counselling in Early-Stage Chronic Kidney Disease Managed in Primary Care: A Non-Randomised Controlled Study
Source: J Clin Med. 2026 Apr 21;15(8):3169. doi: 10.3390/jcm15083169 (PMC13117030; doi:10.3390/jcm15083169)
Supplement: Supplementary file 1 [file jcm-15-03169-s001.zip › Table S3.pdf]

Table S3. Additional descriptive statistics for 3-month changes in outcomes presented in Table 1.

OUTCOME (units)	PA GROUP		COMBINED GROUP		CONTROL GROUP	
	median (25th–75th)	95% CI (Δ mean)	median (25th–75th)	95% CI (Δ mean)	median (25th–75th)	95% CI (Δ mean)
– eGFR (mL/min/1.73 m²)	0.50 (–3.75–5.75)	–2.26, 5.33	1.00 (–2.00–4.00)	–0.75, 4.51	0.00 (–7.00–4.00)	–3.37, 1.81
– ACR (mg/mmol)	0.00 (−0.40−0.06)	–3.68, 1.47	0.00 (−0.70−0.00)	–2.05, 0.33	0.00 (0.00−1.20)	–0.06, 2.42
– sCr (μmol/L)	–0.50 (–8.50–5.00)	–6.54, 3.39	–1.00 (–7.00–4.00)	–5.60, 1.04	0.00 (–6.50–7.50)	–3.25, 4.43
– FPG (mmol/L)	0.00 (–0.20–0.37)	–0.14, 0.55	0.00(–0.70–0.20)	–0.62, 0.01	0.20 (0.00–0.80)	–0.11, 1.68
– HbA1c (%)	0.00 (–0.20–0.00)	–0.42, 0.51	0.00 (–0.28–0.08)	–0.41, 0.13	0.05 (0.00–0.30)	–0.05, 0.52
– Cholesterol (mmol/L)	0.00 (–0.60–0.60)	–0.39, 0.35	0.10 (–0.50–0.20)	–0.45, 0.19	0.00 (–0.30–0.20)	–0.33, 0.38
– LDL (mmol/L)	0.00 (–0.37–0.57)	–0.21, 0.39	0.00 (–0.30–0.30)	–0.28, 0.17	0.00 (–0.25–0.15)	–0.29, 0.30
– HDL (mmol/L)	0.00 (–0.17–0.07)	–0.14, 0.03	0.00 (–0.10–0.10)	–0.09, 0.07	0.00 (–0.10–0.10)	–0.05, 0.09
– TG (mmol/L)	0.00 (–0.40–0.10)	–0.34, 0.10	0.00 (–0.20–0.30)	–0.11, 0.19	0.00 (–0.10–0.10)	–0.24, 0.32
BMI, kg/m²	–0.10 (–0.77–0.15)	–0.54, 0.15	0.00 (–0.60–0.20)	–0.57, 0.11	0.00 (0.00–0.20)	–0.01, 0.23
Chronic diagnoses	0.00 (0.00–0.00)	–0.54, 0.24	0.00 (–1.00–0.00)	–0.73, 0.09	0.00 (0.00–0.50)	0.09, 0.58
Medications	0.00 (–0.75–0.00)	–0.61, 0.22	0.00 (0.00–0.00)	–0.36, 0.44	0.00 (0.00–1.00)	0.07, 1.04
OTC/ supplements	0.00 (0.00–0.00)	–0.36, 0.27	0.00 (0.00–0.00)	–0.77, –0.02	0.00 (0.00–0.00)	–0.08, 0.23
Systolic BP, mmHg	–4.50 (–20.3–0.00)	–15.9, –2.49	–3.00 (–14.0–3.00)	–13.2, –0.05	0.00 (0.00–5.00)	–0.73, 4.66
Diastolic BP, mmHg	–1.00 (–9.25–0.00)	–10.6, –1.18	0.00 (–4.00–3.00)	–9.40, 1.08	0.00 (–1.00–5.00)	–0.47, 4.91

Δ: change from baseline to 3 months (3-month value minus baseline value); PA: physical activity; eGFR: estimated glomerular filtration rate; ACR: albumin-to-creatinine ratio; sCr: serum creatinine; FPG: fasting plasma glucose; HbA1c: haemoglobin A1c; LDL: low-density

lipoprotein; HDL: high-density lipoprotein; TG: triglycerides; BMI: body mass index; OTC: over-the-counter; BP: blood pressure. Values are presented as median (25th–75th percentile) and 95% confidence intervals (CI) for the mean change.
