# Supplementary material for: Short-Term Effects of Structured Physical Activity With or Without Dietary Counselling in Early-Stage Chronic Kidney Disease Managed in Primary Care: A Non-Randomised Controlled Study
Source: J Clin Med. 2026 Apr 21;15(8):3169. doi: 10.3390/jcm15083169 (PMC13117030; doi:10.3390/jcm15083169)
Supplement: Supplementary file 1 [file jcm-15-03169-s001.zip › Table S4-6.pdf]

**Table S4–6. Baseline and 3-month values by study group for SF-36, ISI, and IPAQ.**

**Table S4.** SF-36 domain and summary scores at baseline and after 3 months, by study group.

| SF-36 QUESTIONNAIRE DOMAIN | PA GROUP | | | COMBINED GROUP | | | CONTROL GROUP | | |
|---|---|---|---|---|---|---|---|---|---|
| | baseline | 3-month | p | baseline | 3-month | p | baseline | 3-month | p |
| PF | 55.0 (32.5–68.8) | 60.0 (45.0–70.0) | 0.667 | 50.0 (35.0–75.0) | 60.0 (45.0–75.0) | 0.113 | 50.0 (35.0–67.5) | 45.0 (35.0–67.5) | 0.808 |
| RLP | 50.0 (6.25–87.5) | 62.5 (0.0–100) | 0.594 | 50.0 (25.0–100) | 50.0 (0.0–75.0) | 0.822 | 25.0 (0.0–50.0) | 25.0 (0.0–50.0) | 0.592 |
| RLE | 83.3 (33.3–100) | 100 (33.3–100) | 0.408 | 66.7 (33.3–100) | 100 (66.7–100) | 0.001 | 100 (66.7–100) | 66.7 (33.3–100) | 0.073 |
| E/F | 57.5 (46.3–70.0) | 65.0 (55.0–78.8) | 0.020 | 50.0 (35.0–60.0) | 60.0 (55.0–75.0) | <0.001 | 55.0 (45.0–60.0) | 50.0 (45.0–62.5) | 0.592 |
| EWB | 66.0 (56.0–79.0) | 80.0 (60.0–88.0) | 0.001 | 72.0 (56.0–76.0) | 80.0 (68.0–88.0) | 0.005 | 60.0 (54.0–66.0) | 60.0 (56.0–64.0) | 0.344 |
| SF | 75.0 (56.9–100) | 87.5 (75.0–100) | 0.323 | 75.0 (67.5–87.5) | 87.5 (75.0–100) | 0.007 | 62.5 (55.0–75.0) | 62.5 (55.0–75.0) | 0.528 |
| P | 57.5 (50.0–79.4) | 67.5 (51.9–86.9) | 0.288 | 67.5 (50.0–80.0) | 77.5 (50.0–80.0) | 0.164 | 50.0 (41.3–63.8) | 50.0 (40.0–68.8) | 0.886 |
| GH | 58.0 (50.0–67.0) | 60.0 (50.5–73.5) | 0.097 | 54.0 (42.0–70.0) | 65.0 (52.0–77.0) | 0.017 | 42.0 (36.0–53.5) | 44.0 (35.0–51.5) | 0.416 |
| Summary scores | | | | | | | | | |
| PCS | 47.6 (43.5–54.3) | 51.9 (41.7–55.8) | 0.353 | 45.3 (41.3–54.0) | 50.3 (46.6–57.1) | 0.007 | 48.8 (43.3–51.3) | 46.8 (41.9–51.5) | 0.182 |
| MCS | 49.2 (45.8–56.1) | 50.9 (48.5–58.0) | 0.053 | 51.0 (47.1–57.4) | 55.6 (49.2–58.0) | 0.125 | 46.3 (43.7–50.6) | 46.2 (44.3–49.7) | 0.374 |

SF-36: Short Form-36; PA: physical activity; PF: physical functioning; RLP: role limitations due to physical health; RLE: role limitations due to emotional problems; E/F: energy/fatigue; EWB: emotional well-being; SF: social functioning; P: pain; GH: general health; PCS: Physical Component Summary; MCS: Mental Component Summary. Values are presented as median (25th–75th percentile) with within-group p values (Wilcoxon signed-rank test).

**Table S5.** ISI scores at baseline and after 3 months, by study group.

| ISI QUESTIONNAIRE DOMAIN | PA GROUP | | | COMBINED GROUP | | | CONTROL GROUP | | |
|---|---|---|---|---|---|---|---|---|---|
| | baseline | 3−month | p | baseline | 3−month | p | baseline | 3−month | p |
| SO | 1.00 (0.00−2.00) | 1.00 (0.00−1.75) | 0.312 | 2.00 (1.00−2.00) | 2.00 (1.00−2.00) | 0.667 | 1.00 (0.50−2.00) | 1.00 (0.00−2.50) | 0.572 |
| SM | 1.00 (0.00−2.00) | 1.00 (0.00−1.00) | 0.152 | 2.00 (1.00−2.00) | 1.00 (1.00−2.00) | 0.236 | 2.00 (1.00−2.00) | 2.00 (1.00−2.00) | 0.015 |
| EMA | 1.00 (0.00−2.00) | 1.00 (0.00−1.75) | 0.465 | 1.00 (0.00−2.00) | 1.00 (0.00−2.00) | 0.834 | 1.00 (0.00−2.00) | 1.00 (1.00−2.00) | 0.660 |
| SS | 1.00 (0.25−2.00) | 1.00 (0.00−1.75) | 0.212 | 2.00 (1.00−3.00) | 1.00 (1.00−3.00) | 0.382 | 1.00 (1.00−2.50) | 1.00 (1.00−2.00) | 0.023 |
| DI | 1.00 (0.00−2.00) | 1.00 (0.00−1.00) | 0.209 | 1.00 (1.00−2.00) | 1.00 (0.00−1.00) | 0.155 | 1.00 (0.00−2.00) | 1.00 (0.50−2.00) | 0.407 |
| N | 0.00 (0.00−1.00) | 0.00 (0.00−1.75) | 0.666 | 1.00 (0.00−1.00) | 0.00 (0.00−1.00) | 0.308 | 1.00 (1.00−2.00) | 1.00 (0.50−2.00) | 0.588 |
| D | 0.50 (0.00−1.00) | 1.00 (0.00−1.75) | 0.243 | 1.00 (0.00−2.00) | 0.00 (0.00−1.00) | 0.055 | 1.00 (0.00−2.00) | 1.00 (1.00−2.00) | 0.078 |
| ISI total | 7.00 (1.00−10.80) | 5.00 (1.25−8.75) | 0.537 | 8.00 (6.00−11.00) | 8.00 (4.00−11.00) | 0.092 | 9.00 (5.50−12.50) | 11.00 (6.00−14.50) | 0.250 |

ISI: Insomnia Severity Index; PA: physical activity; SO: sleep onset; SM: sleep maintenance; EMA: early morning awakening; SS: sleep satisfaction; DI: daytime impairment; N: noticeability; D: distress. Values are presented as median (25th–75th percentile) with within-group p values (Wilcoxon signed-rank test).

**Table S6.** IPAQ scores at baseline and after 3 months, by study group.

| IPAQ QUESTIONNAIRE DOMAIN | PA GROUP | | | COMBINED GROUP | | | CONTROL GROUP | | |
|---|---|---|---|---|---|---|---|---|---|
| | baseline | 3−month | p | baseline | 3−month | p | baseline | 3−month | p |
| MET vigorous | 720 (0−1440) | 1080 (0−2880) | 0.026 | 0 (0−640) | 240 (0−1440) | 0.944 | 0 (0−40.0) | 0 (0−80) | 0.206 |
| MET moderate | 440 (0−2160) | 1320 (480−2700) | 0.100 | 240 (0−1680) | 480 (240−1280) | 0.848 | 480 (120−2160) | 240 (0−580) | 0.004 |
| MET walking | 1386 (644−2277) | 1782 (718−2772) | 0.291 | 693 (297−1980) | 990 (594−2079) | 0.835 | 924 (429−1914) | 594 (371−1386) | 0.472 |
| Sitting time | 225 (180−300) | 180 (120−300) | 0.118 | 300 (240−390) | 300 (240−360) | 0.288 | 240 (150−300) | 240 (180−340) | 0.044 |
| MET total | 2664 (1438−4472) | 5151 (3747−6413) | 0.002 | 1752 (873−4320) | 2666 (1695−4212) | 0.686 | 1980 (1223−3724) | 1244 (643−2444) | 0.014 |

IPAQ: International Physical Activity Questionnaire; PA: physical activity; MET: metabolic equivalent. Values are presented as median (25th–75th percentile) with within-group p values (Wilcoxon signed-rank test).
